# A study of extractive summarization of long documents incorporating local topic and hierarchical information

**DOI:** 10.1038/s41598-024-60779-z

**Published:** 2024-05-02

**Authors:** Ting Wang, Chuan Yang, Maoyang Zou, Jiaying Liang, Dong Xiang, Wenjie Yang, Hongyang Wang, Jia Li

**Affiliations:** 1 School of Computer Science, Chengdu University of Information Technology, Chengdu, 610225 Sichuan Province China; 2 College of Blockchain Industry, Chengdu University of Information Technology, Chengdu, 610225 Sichuan Province China

**Keywords:** Computational science, Computer science

## Abstract

In recent years, the transformer-based language models have achieved remarkable success in the field of extractive text summarization. However, there are still some limitations in this kind of research. First, the transformer language model usually regards the text as a linear sequence, ignoring the inherent hierarchical structure information of the text. Second, for long text data, traditional extractive models often focus on global topic information, which poses challenges in how they capturing and integrating local contextual information within topic segments. To address these issues, we propose a long text extractive summarization model that employs a local topic information extraction module and a text hierarchical extraction module to capture the local topic information and document's hierarchical structure information of the original text. Our approach enhances the ability to determine whether a sentence belongs to the summary. In this experiment, ROUGE score is used as the experimental evaluation index, and evaluates the model on three large public datasets. Through experimental validation, the model demonstrates superior performance in terms of ROUGE-1, ROUGE-2, and ROUGE-L scores compared to current mainstream summarization models, affirming the effectiveness of incorporating local topic information and document hierarchical structure into the model.

## Introduction

Text summarization is an arduous task in the field of natural language processing (NLP)^1^, wherein the goal is to generate a concise and logically connected summary of a given document. This process involves extracting crucial information and reduce the length of the document while preserving the essential meaning^2,3^. Text summarization can effectively reduce the information burden of users, enable users to quickly obtain information from redundant information, greatly reduce manpower and material resources. It plays an important role in various domains, including information retrieval, title generation and other related fields.

Based on the methodology employed, text summarization tasks can be categorized into two types: extractive summarization^4^ and abstractive summarization^5^. The abstractive summarization method utilizes neural network-based approaches, such as the Sequence-2-Sequence (Seq2Seq) architecture^6^, also known as encoder-decoder architecture. The principle of an encoder-decoder is similar to the way human think or write summaries. The encoder first encodes the full text, and then the decoder generates new sentences word by word to form a document summary. This method generates less redundant summary information, but might face challenges in maintaining fluency and grammatical correctness. In addition, the generation of new words or phrases may produce summaries that are inconsistent with the original statement^7^. These issues can be mitigated by directly selecting sentences from the source text and assembling them into summaries, i.e. the extractive summarization. The extractive method treats summarization as a classification problem, where important sentences are directly selected from the source text to construct a summary. Summaries generated through this approach often exhibit a good performance in fluency and grammar. For the extractive summarization task, the core challenge lies in learning comprehensive sentence context information and modeling inter-sentence relationships through the encoder, thereby enabling sentence classifiers to extract more valuable sentences. Traditional extractive methods usually employ graph-based methods or clustering-based methods for unsupervised summarization^8,9^. These approaches construct the correlation between sentences using cosine similarity, and then use sorting methods to calculate the importance of sentences. With the rapid development of deep learning, many extractive summarization methods use Recurrent Neural Network (RNN) to capture the relationship between sentences^10,11^. However, RNN-based methods are difficult to deal with long-distance dependencies, especially for long document summaries. In recent years, transformer^12^ language model, which has been pre-trained by large-scale corpus, has achieved excellent results when fine-tuned for downstream tasks, and have found widespread application in the field of text summarization. Liu et al. ^13^ proposed the BERTSUM model by improving the BERT embedding layer. They applied the BERT model for the first time in the text summarization and achieved state-of-the-art (SOTA) performance on CNN/DailyMail dataset. Zhang et al. ^14^ designed a hierarchical transformer to capture long-range inter-sentence relationships. However, this method did not yield significant performance gains for summarization tasks and faced challenges such as slow training speed and potential overfitting. At the same time, some researchers introduced the neural topic model (NTM)^15^ and graph neural network (GNN)^16^ into the task of text summarization to capture global semantic information and further guide the generation of abstracts. Cui et al. ^17^ use NTM to capture the theme features of documents and GNN to represent documents as graph structures, thus obtaining the relationship between sentences.

However, for long document summarization tasks, the above methods have two shortcomings. The first one is that they fail to recognize the explicit hierarchical structures and section headings inherent within the long document. When manually summarizing text, we tend to focus on the main sections. For example, in the context of scientific papers, more attention may be given to sections like "Methodology", "Experimental" and "Conclusion", but "Background" or "Related Work" may not receive as much emphasis. In addition, sentences within a section have stronger relationships compared to those outside the section. Understanding the logical relationship between sentences and the hierarchical structure within the document helps the model better identify the important sentences. However, the traditional transformer-based text summarization methods often regard the text as a sequential structure, and struggle with longer documents. The second shortcoming is that the longer the document, the more topics it may discuss, because each section presents different topic information. In summary, the aforementioned methods focus on the overall topic information of the entire document, that is, the global information, neglecting the local topic information of individual sections. In order to address these issues, this paper proposes a long-document extractive summarization model that integrates local topic information and document hierarchy information into current topic segment.

The main contributions of this paper can be summarized as follows:

1. Introduction of an innovative long-document extractive summarization model. This model consists of a text encoder, a module for extracting local topic information, and a module for embedding hierarchical structure information of the document. The information is integrated into the sentence representation of the document, enhancing the quality of the generated summaries.
2. This paper utilizes LSTM-Minus^18^ to obtain distributed representations of local information and combines it with text summarization tasks. Instead of employing a fixed three-segment approach for text paragraphing, the paper adopts a dynamic method based on the number of sentences to determine paragraph length, thereby calculating the starting and ending positions of each paragraph in the text. Paragraph segments are divided based on these positions, and their topic information is computed.
3. Experimental results conducted on the PubMed dataset reveal excellent performance of the proposed method when compared to several baseline models.

## Related Work

### Extractive summarization method

With the rapid development of neural networks, significant achievements have been made in extractive summarization tasks. At present, the extractive methods are mainly regarded as sentence sorting task or binary sequence labeling tasks. In the sentence sorting paradigm, models are required to assign scores to each sentence in the text and place higher-scored sentences at the front of the summary list while lower-scored sentences are placed towards the back. This process yields an ordered list of sentences, and the top few sentences are selected as the summary. Narayan et al. ^19^ proposed a topic-aware convolutional neural network model. This model first extracts features from the documents using convolutional neural networks and then weights the features according to the topic. Finally, a selection-based sorting method is employed to choose the most relevant sentences as the summary. Experiments results on multiple datasets show that this approach can generate concise summaries that still preserve valuable information. Li et al.^20^ proposed a method for evaluating sentence importance in multi-document summarization using variational autoencoder. Different from the traditional method based on feature engineering, this method directly learns the abstract semantic representation directly from the original data. KL divergence is introduced to constrain the generated sentence representations to be close to the prior distribution, thereby improving the generalization ability of the model.

Regarding the second paradigm, which considers extractive text summarization as a sequence labeling task, this approach involves extracting and encoding features for each sentence or paragraph. The encoded features are then input into a decoder for labeling prediction to determine which sentences should be selected for the summary. The sequence labeling method has been widely applied in extractive text summarization and has achieved good results. Nalapati et al.^4^ proposed the SummaRuNNer model for text summarization, which is a sequence model based on RNN. This model generates document summarization by learning the importance of each sentence within the document. It has demonstrated good summarization performance on multiple text datasets. Zhang et al.^21^ introduced a latent variable extractive model, which treats sentences as latent variables and infers summaries using sentences with activated variables.

However, most of the methods mentioned above rely on RNN for extractive summarization. RNN-based methods face challenges in handling long-distance dependencies at the sentence level and may omit on language or structural information due to the input format of the original document. In order to address these issues, researchers have started utilizing transformer-based pre-training language model as encoders and representing documents through more intuitive graph structures. They have also incorporated NTM to extract topic features from the documents, further guiding the models to produce high-quality summaries. Jia et al.^22^ proposed a method called deep differential amplifier for extractive summarization, which enhances the features of summary sentences by contrast to non-summary sentences using differential amplifiers. Shi et al.^23^ proposed a star architecture-based extractive summarization method, where sentences in documents are modeled as satellite nodes, and a virtual central node is introduced to learn the inter-sentence relationships using the star structure. This approach achieved promising results on three public datasets. Ma et al.^24^ embedded the topic features extracted by NTM into BERT to generate a vector representation with topic features, thus improving the quality of summaries.

Although the aforementioned methods have succeeded in modeling inter-sentence relationship and extracting global semantics, there is still a problem with extractive text summarization methods based on transformer pre-training language models. The length of the input document in text summarization is longer compared to general natural language processing task, and using just a transformer-based encoder is insufficient for effectively handling long texts and often leads to high computational costs. To better understand the original document, researchers have proposed various improvements. Xie et al.^25^ first preprocessed the documents by dividing them into blocks with the same size, encoded each block with block encoding. They merged the block encoding results with NTM to generate global topic features. Finally, they established a comparison graph between topic features and sentence features to filter summary sentences. This method has achieved good results in both long documents and short news documents, with particular advantages in handling the former. Beltagy et al.^26^ introduced the Longformer model, specifically designed for processing long documents. By replacing the self-attention mechanism of the transformer with a sliding window self-attention mechanism, the time complexity is reduced to linear level, enabling the model to handle long documents easily. Although the Longformer performs well in handling long documents, it fails to model local semantic information and document hierarchy structure, which affects its performance. Therefore, this paper uses the Longformer as the encoder and incorporates local contextual information of the current topic segment and hierarchical structure information of the document. This allow our model to prioritize local topic information and overall structural information when dealing with long scientific papers.

### LSTM-Minus

Wang et al.^27^ proposed the LSTM-Minus method for the first time, and applied it to dependency parsing and achieved good results. The LSTM-Minus method is a novel approach for learning embedding of text segments, utilizing subtraction between LSTM hidden vectors to learn the distributed representation of sentence segments. Initially, a sentence is divided into three segments (prefix, infix and suffix), and the segment from the word ${w}_{i}$ to the word ${w}_{j}$ is represented by the hidden vector ${h}_{j}-{h}_{i}$. This allows the model to effectively learn segment embedding from both external and internal information, thus enhancing its ability to obtain sentence-level information. In the same year, Cross et al.^28^ extended the unidirectional LSTM-Minus to the bidirectional, using it as sentence span representation and achieving impressive performance in component syntactic analysis tasks. Build upon this idea, we applied this method to the field of text summarization to extract the contextual information from local topic segments.

## Method

To address the limitations of the existing extractive text summarization methods, this paper proposes a long document extractive summarization model that integrates local contextual information and document-level hierarchical information from the current topic segment. The model is inspired by the long document extractive model proposed by Ruan et al.^29^, which incorporates hierarchical structure information. The final model of this paper is obtained by incorporating local topic information. Experiments results show that the inclusion of local topic information further deepens the model's understanding of long texts. The task of long text extractive summarization is defined as: follow: Given an original document ${\text{D}}=\{{sent}_{1},\dots ,{sent}_{n}\}$, D contains n sentences, where each sentence denoted as the ${sent}_{i}$, represents the i-th sentence of the original document. The purpose of the extractive text summarization model is to select m sentences capturing the central idea of the original text as summaries, where m is the desired number of summary sentences $\left(m\ll n\right)$. This task is typically treated as a sentence classification problem. For each sentence ${sent}_{i}$, there is a corresponding label ${y}_{i}\in \left\{\mathrm{0,1}\right\}$, where a label of 1 means that the sentence belongs to the summary, while 0 indicates that it does not.

**Figure 1:**
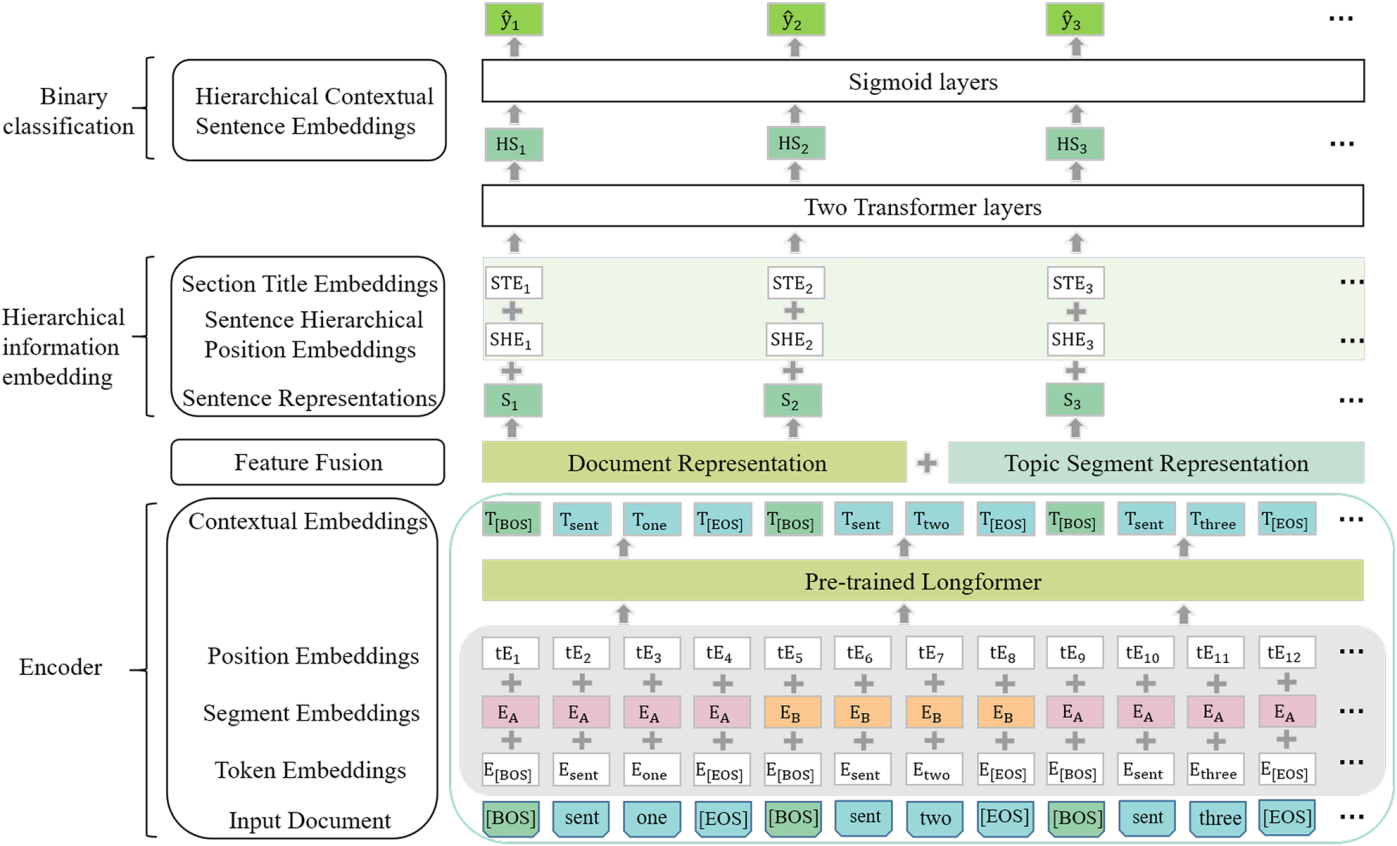
Overall structure diagram of the model.

**Figure 2:**
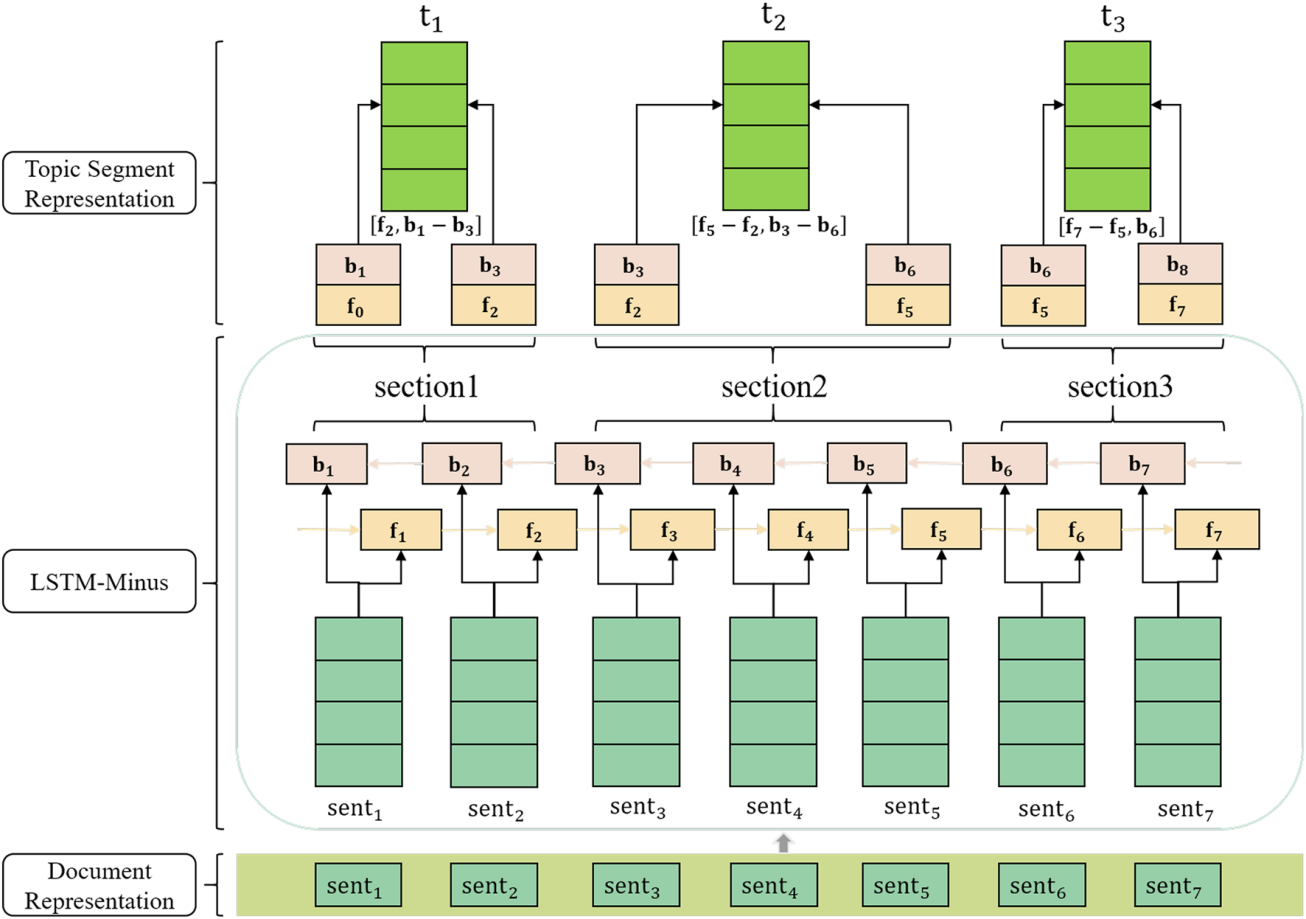
Local topic information extraction module.

The proposed model, as shown in Fig. 1, comprises three main modules: a pre-trained language model based encoder, a local topic information extraction module (referred to as the Topic Segment Representation module in the Fig. 1), and a text hierarchical information embedding module. Because this work deals with long text corpus, the encoder used is based on the Longformer, an improvement over the transformer pre-training language model, which allows for better encoding of long documents. Once the contextual representation of the document is obtained through the encoder, it is passed to the local topic information extraction module, which extracts the topic information of the sentence segment it belongs to. The specific structure of this module is shown in Fig. 2. Then, the local topic information representation is fused with the text contextual representation, resulting in a fusion of the local topic information and the textual context. The text hierarchical structure information embedding module embeds the hierarchical structure information of the text into the fused representation of the local topic information and textual context. By using a two-layer stacked transformer, this module learns the hierarchical structure information at both the sentence and document levels, enabling the model to gain a deeper understanding of the text context. Finally, the confidence score of each sentence is calculated through Sigmoid layer for each sentence to determine whether it should be included in the summary.

### Text hierarchical information

#### Sentence hierarchical information

Due to scientific papers consisting of multiple sections, with each section containing several paragraphs that describe different topics, this paper uses paragraphs as the unit for hierarchical division of the article. The sentence-level hierarchical structure information includes the linear position of the paragraph to which the sentence belongs and the linear position representation of the sentence within the paragraph. The positions of paragraphs and sentences are represented by numerical serial numbers corresponding to them. For an original document ${\text{D}}=\left\{{sent}_{1},\dots ,{sent}_{n}\right\}$, the hierarchical structure information of the i-th sentence ${sent}_{i}$ is expressed as a two-dimensional vector $\left({s}_{s},{s}_{g}\right)$, which indicates the position of the sentence at this level, as shown in Formula (1).

1 $${vsent}_{i}=({s}_{s},{g}_{s})$$

where ${s}_{s}$ represents the linear position of the paragraph containing the sentence relative to the entire article, and ${g}_{s}$ represents the linear position of the sentence within its respective paragraph. All sentences within the same paragraph share the same value in the first dimension of the $vsent$ vector, indicating a higher correlation among sentences within the same paragraph. And the ${g}_{s}$ vector further indicates the linear relationship among sentences within the paragraph.

#### Section title information

Compared with short news articles, scientific papers often have section titles. The content within each section is usually highly relevant to the corresponding section title, as the section title serves as a concise summary of the content of the section. In this study, when encoding sentences, the section titles are incorporated as additional hierarchical information into the sentence encoding. However, for scientific papers, there are many similar section titles with the same meaning. For instance, "Method" and "Methodology" have similar meanings and can be grouped together under the "Method" category. Therefore, for the PubMed dataset used in this paper, eight section title categories are defined^29^, including “introduction”, “background”, “case”, “Method”, “result”, “discussion”, “conclusion”, and “additional information”. If the section title of a section does not fall into any of the eight predefined categories, the original section title itself is directly used.

### Encoder

#### Document encoding

The purpose of document encoding is to encode the sentences of the input document into a vector representation with a fixed length. Previous methods for extractive text summarization tasks often employed RNN and BERT^30^ as encoders. BERT is a bidirectional transformer encoder that is pre-trained on large-scale corpus and has achieved excellent performance on various natural language processing tasks. However, for long text data, BERT cannot process the entire document, which will lead to information loss. Therefore, in this paper, we uses the Longformer pre-training language model as the text encoder. Longformer improves the self-attention mechanism of the traditional transformer into the sliding window self-attention, which makes it easy to handle documents with thousands of characters. In the traditional transformer self-attention mechanism, the calculation is performed by linearly transforming the input word embedding matrix to generate a Query matrix (Query, Q), a Key matrix (Key, K), and a Value matrix (Value, V) of dimension d. The specific calculation process is shown in Formula 2.

2 $${\text{Attention}}={\text{softmax}}\left(\frac{Q{K}^{T}}{\sqrt{{d}_{k}}}\right)V$$

where $\left(Q,K,V\right)\in {R}^{L\times d}$, and d represents the dimension of a word vector matrix, while ${d}_{k}$ represents the dimension of the K matrix. Hence, the spatial complexity of the traditional transformer self-attention mechanism is ${\text{O}}\left({L}^{2}\right)$, the spatial complexity of Longformer’s sliding windows self-attention mechanism is ${\text{O}}\left(L\right)$, scaling linearly with the input sequence length L. As a result, Longformer has more advantages in encoding long texts.

As shown in Fig. 1, in order to obtain the representation of each sentence, we inserts [BOS] (beginning of sentence) and [EOS] (end of sentence) tags at the beginning and end of each sentence respectively. The model embedding layer includes Token Embeddings (TE), Segment Embeddings (SE) and Position Embeddings (PE). These features are sum to obtained the embedded representation of each word. Subsequently, the context of the input sequence is learned by using the pre-trained Longformer. The entire procedure is illustrated in Eqs. (3) and (4).

3 $${w}_{i,j}=\left(TE+SE+PE\right)$$

4 $$\left\{{h}_{\mathrm{1,0}},{h}_{\mathrm{1,1}},\dots ,{h}_{N,0},\dots ,{h}_{N,*}\right\}=\mathrm{ Longformer}\left({w}_{\mathrm{1,0}},{w}_{\mathrm{1,1}},\dots ,{w}_{N,0},\dots ,{w}_{N,*}\right)$$

where ${w}_{i,j}$ represents the j-th word of the i-th sentence, which is obtained by Formula 3. ${w}_{i,0}$ and ${w}_{i,*}$ represent the [BOS] and [EOS] tags of the i-th sentence respectively, and ${h}_{i,j}$ represents the hidden state of the corresponding word. After Longformer encoding, we use the [BOS] tag as the context representation of each sentence, that is,${H}_{s}=\left({h}_{\mathrm{1,0}},\dots ,{h}_{N,0}\right)$.

#### Local topic information extraction

In order to capture the local context information of the text segment to which the sentence belongs, this study employs the LSTM-Minus method to learn text segment embeddings. Its detailed structure is shown in Fig. 2. The input of the local topic information extraction module is the contextual representation of each sentence obtained by the encoder. LSTM can learn and utilize the previous information through its own gating structure and store it in the memory cell. Therefore, this study utilizes a Bi-LSTM to encode the sentence context representations to get the hidden vector representation of each sentence. Subsequently, the local contextual information of the topic segment to which the sentence belongs is represented by the subtraction between the hidden vectors at the beginning and end of each topic segment. For the i-th topic segment ${t}_{i}$, the specific expression method can be found in Eqs. (5), (6), (7).

5 $${f}_{i}={h}_{{end}_{i}}^{f}-{h}_{{start}_{i-1}}^{f}$$

6 $${b}_{i}={h}_{{start}_{i}}^{b}-{h}_{{end}_{i+1}}^{b}$$

7 $${t}_{i}=\left({f}_{i}|{b}_{i}\right)$$

where ${f}_{i}$ and ${b}_{i}$ respectively represent the forward and backward propagating topic segments, while ${start}_{i}$ and ${end}_{i}$ respectively represent the starting position and ending position of the topic segment. “|” represents the vector concatenation symbol. In the case of the second topic segment ${t}_{2}$ in Fig. 2, the local topic information can be expressed as $\left[{f}_{5}-{f}_{2},{b}_{3}-{b}_{6}\right]$, where ${f}_{5}$ and ${f}_{2}$ represent the forward propagating hidden state of the fifth sentence and the second sentence respectively, and ${b}_{3}$ and ${b}_{6}$ represent the backward propagating hidden state of the third sentence and the sixth sentence respectively. To prevent index out of range, this study introduces zero vectors at the beginning and end of both forward and backward propagation respectively. After calculating the local contextual information of the topic segment to which the sentence belongs, it is concatenated with the sentence context encoding of the document to further enrich the sentence contextual representation.

#### Text hierarchical information encoding

Currently, there are two mainstream linear position encoding methods. The first one, used in transformer^12^, involves generating fixed values using sine/cosine functions. The second one, used in BERT^30^, involves generating random values that are trainable. The position encoding method in transformer can only mark the position of a character, without considering its contextual information. On the contrary, BERT's position encoding is achieved by randomly initializing an embedding vector with the dimension of $\left[seq\_length,width\right]$. Therein, the first dimension represents the sequence length, and the second dimension represents the vector length corresponding to each character. They are trained along with the entire extractive model, allowing it to not only mark the character position, but also learn the function of this position. Therefore, this study uses BERT position encoding method to encode $vsent$ vector. The hierarchical structure vector $\left({s}_{s},{s}_{g}\right)$ of the i-th sentence can be expressed as Formula (8).

8 $${SHE}_{i}=PE\left({s}_{s},\frac{d}{2}\right)|PE\left({g}_{s},\frac{d}{2}\right)$$

where *PE* represents the position encoding method of BERT, and *d* represents the vector dimension of the sentence, while “|” represents the vector concatenation symbol.

In order to encode the section title information (STE) of a sentence, this study uses the same pre-trained encoder for document encoding. By inputting the extracted section titles into the pre-trained encoder, the hidden states corresponding to each character are obtained and then summed up. This approach allows for better integration of semantic information from each position within the section title, in order to provide a more comprehensively representation of the section title information.

### Training and Infer

After obtaining the output sentence vector from the text hierarchical structure information embedding module, a two-layer stacked transformer is applied to learn the hierarchical information at the sentence and document levels. Subsequently, these vectors are input into a sigmoid function to predict whether a sentence belongs to the summary. In the training stage, this model uses binary cross entropy as the loss function, aiming to minimize the binary cross entropy loss function to optimize the model. See Eqs. (10) and (11) for details.

9 $${\widehat{y}}_{i}=\sigma \left({W}_{h}\cdot {HS}_{i}+{b}_{h}\right)$$

10 $${\text{Loss}}=\left\{{loss}_{1},\dots ,{loss}_{n}\right\}$$

11 $${loss}_{i}\left({\widehat{y}}_{i},{y}_{i}\right)=-\left[{y}_{i}*log\left({\widehat{y}}_{i}\right)+\left(1-{y}_{i}\right)*log\left(1-{\widehat{y}}_{i}\right)\right]$$

where, $\upsigma$ indicates sigmoid function, ${W}_{h}$ represents learnable parameter matrix, ${HS}_{i}$ represents the sentence vector representation that incorporates local topic information and hierarchical structure information, ${b}_{h}$ represents bias. In Formula 10, ${loss}_{i}$ represents the loss when judging whether each sentence belongs to summary, ${\widehat{y}}_{i}$ represents the predicted probability value of the current sentence, and ${y}_{i}$ represents the true label value of the sentence.

## Experiment

### Dataset

In order to verify the generalization ability of the model in this article, experiments were conducted on three datasets, namely the short news text dataset CNN/Daily Mail, and two long text datasets PubMed and ArXiv. The CNN/Daily Mail dataset comprises 310,000 news articles. The PubMed dataset is generated based on the PubMed literature database, which provides a search engine for biomedical literature. The other long text dataset is ArXiv, which contains papers from various domains. Table 1 provides a detailed comparison of the document count, average text length, and average summary length of the CNN/Daily Mail dataset, PubMed dataset, and ArXiv dataset.

**Table 1:** Comparison of lengths of three datasets.

Datasets	Number of documents	Average document length (words)	Average abstract length (words)
CNN	92,579	656	43
DailyMail	219,506	693	52
PubMed	133,215	3016	203
ArXiv	215,913	5825	272

As shown in Table 2, following Cohan et al.^31^ and See et al.^11^, the training, testing, and validation set sizes for the PubMed dataset, ArXiv dataset, and CNN/DM dataset are presented, respectively.

**Table 2:** Division of three datasets.

Dataset	Train	Test	Val
CNN/DM	287,227	11,490	13,368
PubMed	119,924	6633	6658
ArXiv	203,037	6440	6436

### Evaluation metrics

In this paper, ROUGE^32^ (Recall-Oriented Understudy for Gisting Evaluation) score is used to evaluate text summarization models, including ROUGE-L, ROUGE-N, ROUGE-W and ROUGE-S. ROUGE-L is calculated using the longest common subsequence and measures the similarity between the generated summary and the reference summary. ROUGE-N (where N can be 1, 2, 3, or 4) is an evaluation method based on n-gram recall rate. The fundamental idea is to calculate the co-occurrence information score between the model-generated summary and the manually generated reference summary to assess the similarity between them. In this paper, ROUGE-1 (R-1), ROUGE-2 (R-2) and ROUGE-L (R-L) are used as evaluation metrics, and the specific calculation processes of ROUGE-N and ROUGE-L are shown in Formulas (12), (13), (14), (15).

12 $$ROUGE{-}N = \frac{{\mathop \sum \nolimits_{{S \in \left\{ {{\text{Re}} fSum} \right\}}} \mathop \sum \nolimits_{{gram_{n} \in S}} Count_{match} \left( {gram_{n} } \right)}}{{\mathop \sum \nolimits_{{S \in \left\{ {{\text{Re}} fSum} \right\}}} \mathop \sum \nolimits_{{gram_{n} \in S}} Count\left( {gram_{n} } \right)}}$$

13 $${R}_{lcs}=\frac{LCS\left(RefSum,ModelSum\right)}{m}$$

14 $${P}_{lcs}=\frac{LCS\left(RefSum,ModelSum\right)}{n}$$

15 $$ROUGE{-}L = \frac{{\left( {1 + \beta^{2} } \right)*R_{cls} *P_{cls} }}{{R_{cls} + \beta^{2} P_{cls} }}$$

where,${Count}_{match}\left({gram}_{n}\right)$ indicates the number of n-grams that simultaneously appear in both the generated summary by an article model and the reference summary. ${\text{Count}}\left({gram}_{n}\right)$ donates the number of n-grams in the reference summary. ${\text{LCS}}\left(RefSum,ModelSum\right)$ represents the longest common subsequence of the reference summary and model-generation summary. m represents the length of reference summary, while n represents the length of model-generation summary. $\upbeta$ is a hyper-parameter used in the evaluation metrics.

### Experimental setup

This model was built using the Pytorch deep learning framework and trained on a RTX4090 GPU with 24GB of memory. The training process employed gradient accumulation every two steps. In the experiment, the "Longformer-base-4096" model was chosen as the encoder. Similar to BERT, it consists of 12 layers of transformer encoding with a hidden size of 768. We adopt the same training strategy as Liu et al.^13^, including a warm-up phase for 10,000 steps, followed by training for 50,000 steps. Specific parameters can be found in Table 3.

**Table 3:** Hyperparameter setting.

Hyperparameter	Hyperparametric meaning	Superparameter setting value
Optimizer	optimizer	Adam
Adam_epsilon	Adam Fuzzy shadow	1e-8
${\beta }_{s}$	Beat1and Beta2 values	(0.9,0999)
Batch size	Bath size	600
Learning rate	Learning rate	2e-3
Ext_layer	Stack Transformer layers	2
Ext_dropout	Stacked Transformer dropout	0.1

### Ethical and informed consent

The data utilized in our study are exclusively sourced from publicly available and published datasets. There is no conflict with others’ data, and all data sources have been used in accordance with their respective terms of use and copyright policies.

## Results and analysis

To validate the effectiveness of the proposed model in the field of extractive summarization for long texts, experiments were conducted on publicly available datasets, including PubMed, ArXiv, and CNN/DM. A systematic comparison was performed between the proposed model and recently proposed extractive summarization models as well as abstractive summarization models.

- BERTSUM^13^: This model was the first to introduce BERT to text summarization. It adds a [CLS] token as a sentinel to represent the sentence. During extractive summarization, it predicts scores for each sentence to determine whether they belong to the summary.
- Sent-CLF and Sent-PTR^33^: Both of them use a hierarchical bidirectional LSTM with word and sentence-level representation as encoders. The difference lies in how they determine if a sentence is part of the summary. Sent-PTR uses sentence pointers, while Sent-CLF predicts sentence score.
- Longformer-Ext^26^: This model improves upon transformer-based encoders by introducing a revised attention mechanism that combines local and global attention patterns. It stacks 12 layers of enhanced transformers as the encoder and predicts scores to select summary sentences.
- Reformer-Ext^34^: This model replaces the attention mechanism of the original transformer with a hash-based attention mechanism and incorporates reversible computation. These modifications allows it to handle long text summarization effectively.
- ExtSum-LG + RdLoss^35^ and ExtSum-LG + MMR^35^: Both models utilize ExtSum-LG as the base model. The former adds redundant loss items to the original loss function to minimize redundant sentences during the sentence scoring stage, resulting in summaries with less redundancy. The latter recalculates the sentence importance scores using the obtained sentence confidence scores and selects sentences with lower redundancy as the summary.
- PEGASUS^36^ and T5^37^: The former adopts an unsupervised approach for pre-training, focusing on the task of text summarization. The latter, on the other hand, serves as a Transformer-based general text transformation model.
- TextRank^38^: Mihalcea et al., drawing upon the PageRank algorithm, proposed a methodology utilizing words, phrases, and sentences as nodes, with their relationships represented as edges to construct a graph. This approach facilitates the exploration of relationships among various vertices and edges in the context of their study.
- Topic-GraphSum^39^: Integrating pre-trained language models with topic modeling for the purpose of abstract generation.
- Pointer-Generator + Coverage^11^: Utilizing a pointer generator to directly copy words from the source text while retaining the capability to generate new words. Additionally, employing a coverage mechanism to control the repetition of content in the summary.
- HIBERT^14^: Adopting a Transformer-based bidirectional encoder for document encoding and leveraging unlabeled data for pre-training.
- HSSAS^40^: By introducing a hierarchical self-attention mechanism to encode sentences and documents, the extraction of sentences as summaries is facilitated.

### Analysis of comparative experimental results on the PubMed dataset

According to the experimental results in Table 4, the first two models refer to the unsupervised LEAD model and the greedily constructed ORACLE method. Given that the PubMed dataset consists of lengthy texts, this study employed the LEAD-7 method, extracting the initial 7 sentences as summaries; however, the results were not satisfactory. This observation suggests that the initial sentences in the PubMed dataset do not contain as much information as those in the CNN/DailyMail dataset. The ORACLE summaries are generated using a greedy strategy, selecting sentences that maximize the ROUGE scores and are often considered the upper limit of model performance on this dataset. Through comparative experiments with recent extractive and abstractive summarization models, the proposed long-text extractive summarization model, which combines local topic information and hierarchical structure information, achieves higher R-1, R-2, and R-L scores on the PubMed dataset than other models. This substantiates the effectiveness of our model in comparison to existing approaches.

**Table 4:** Comparison of experimental results on PubMed dataset. Significant values are in bold.

Dataset		PubMed	
Metrics Models	R-1	R-2	R-L
LEAD-7	37.95	13.33	34.10
ORACLE	58.15	34.16	51.69
**Abstractive**			
PEGASUS(2020)	45.49	19.90	**42.42**
BigBird PEGASUA(2020)	46.32	**20.65**	42.33
T5(2020)	9.37	3.70	8.49
**Extractive**			
BERTSUMEXT(2019)	41.09	15.51	36.85
Sent-CLF(2020)	45.01	19.91	41.16
Sent-PTR(2020)	43.30	17.92	39.47
Reformer-Ext(2020)	42.32	15.91	38.26
Longformer-Ext(2020)	43.75	17.37	39.71
ExtSum-LG + RdLoss(2021)	45.30	20.42	40.95
ExtSum-LG + MMR(2021)	45.39	20.37	40.99
**Our Model**	**46.49**	20.52	42.06

Specifically, the model in this article is better than the PEGASUS model in R1 and R2 scores, but the RL is slightly lower. Compared to the BigBird PEGASUS model, R2 and RL are slightly lower than this model, but R1 is slightly higher. Since this article uses the PEGASUS-Large version, the model parameters are 540 M, while the model parameters of this article are only 193 M. It can be seen that the model of this article shows strong performance. Since the T5 model is not specifically trained for text summarization tasks, it shows poor results. Compared to the BERTSUM model, our proposed model shows improvements of 5.4%, 5.01%, and 5.21% in R-1, R-2, and R-L scores, respectively. This indicates that our extractive summarization model, incorporating Longformer as the text encoder, effectively addresses the challenges posed by the length limitations of BERT pre-trained language models. Additionally, when compared to the Longformer-Ext extractive model using Longformer as the encoder, our model achieves improvements of 2.74%, 3.15%, and 2.35% in R-1, R-2, and R-L scores, respectively. This suggests that our proposed approach, combining local context and hierarchical structure information, can effectively enhance the performance of long-text extractive summarization models.

### Analysis of comparative experimental results on ArXiv dataset

Based on the experimental results presented in Table 5, it is evident that, due to the longer length of text in the ArXiv dataset compared to the PubMed dataset, employing the LEAD-10 method to extract the initial 10 sentences as summaries still yields unsatisfactory results. The second section of the table compares the proposed model with generative summarization, while the third section compares it with recent extractive summarization models. Our model demonstrates excellent performance, indicating that the ArXiv dataset exhibits a noticeable hierarchical structure. The introduced hierarchical structure information extraction module in our model proves beneficial in aiding the model's understanding of the source text, thereby enhancing the quality of the generated summaries. Given the significantly longer average length of documents in the ArXiv dataset compared to PubMed, the PEGASUS model's performance on this dataset is slightly lower than its performance on the PubMed dataset.

**Table 5:** Comparison of experimental results on ArXiv dataset.

Dataset		ArXiv	
Metrics models	R-1	R-2	R-L
LEAD-10	37.37	10.85	33.17
ORACLE	53.88	23.05	44.90
Abstractive			
Topic-GraphSum(2021)	44.03	18.52	32.41
PEGASUS(2020)	44.70	17.27	25.80
Extractive			
BERTSUMEXT(2019)	41.24	13.01	36.10
Sent-CLF(2020)	34.01	8.71	30.41
Sent-PTR(2020)	42.32	15.63	38.06
Reformer-Ext(2020)	43.26	14.68	38.10
ExtSum-LG + RdLoss(2021)	44.01	17.79	39.09
ExtSum-LG + MMR(2021)	43.87	17.50	38.97
**Our Model**	**45.84**	**19.03**	**40.36**

Significant values are in bold.

### Analysis of comparative experimental results on CNN/DM dataset

According to Table 6, the unsupervised LEDA-3 performs better on the CNN/DM dataset compared to the PubMed and ArXiv datasets. This is attributed to the nature of CNN/DM as a news-oriented short-text dataset, where the first 3 sentences often encapsulate the majority of the textual meaning. Moreover, in terms of the Rouge-1, Rouge-2, and Rouge-L evaluation metrics, our proposed model exhibits improvements of 4.17, 3.33, and 4.62, respectively, over the TextRank model on the CNN/DM dataset. This suggests that our model outperforms TextRank by considering hierarchical structure information and paragraph-level topic information, while TextRank focuses solely on the similarity between words and sentences. Analyzing the second and third sections of the table, our model competes favorably with PEGASUS and surpasses all other comparative models. The introduced local topic information extraction module and hierarchical structure information in our model offer a more comprehensive representation of the topic ideas in news text, complementing the Transformer encoder and thereby enhancing the quality of the generated summaries.

**Table 6:** Comparison of experimental results on CNN/DM dataset.

Dataset		CNN/DM	
Metrics models	R-1	R-2	R-L
LEAD-3	40.24	17.70	36.45
ORACLE	56.22	33.74	52.19
Abstractive			
Pointer-generator + coverage(2017)	39.53	17.28	36.38
BERTSUMABS(2019)	41.72	19.39	38.76
PEGASUS(2020)	44.17	**21.47**	**41.11**
Extractive			
TextRank(2004)	40.20	17.56	36.44
BERTSUMEXT(2019)	43.25	20.24	39.63
HIBERT-base(2019)	42.31	19.87	38.78
HIBERT-large(2019)	42.37	19.95	38.83
HSSAS(2018)	42.30	17.80	37.60
Reformer-Ext(2020)	38.85	16.46	35.16
Longformer-Ext(2020)	43.00	20.20	39.30
Our Model	**44.37**	20.89	41.06

Significant values are in bold.

### Ablation experiment

In order to validate the effectiveness of incorporating local topic information and text hierarchical structure information in long text extractive summarization tasks, we conducted ablation experiments. Our baseline model shares the same structure as BERTSUM but uses Longformer-base-4096 as the encoder. However, this version can only handle the maximum length of 4096 tokens. To overcome this limitation, we replicated the Token Position Embeddings(TPE) of the original Longformer multiple times until reaching our desired length and subsequently trained additional TPEs with the entire summarization model. Specifically, Local Information (L-Inf) stands for the local topic information, while article hierarchical embeddings (AHE) encompass the embedding of chapter titles and sentence-level structure within the article.

The results of the ablation experiments are shown in Table 7. Compared to the baseline model, adding article hierarchical information improved the model's scores by 4.84%, 4.36%, and 4.57% for R-1, R-2, and R-L, respectively. This indicates that incorporating the hierarchical structure information of texts enables the model to better identify important sentences for long texts. By adding local topic information to the baseline model, we observed score improvements of 4.38%, 3.86%, and 4.15% for R-1, R-2, and R-L, respectively. This suggests that in long text data, different chapters represent different topics, and incorporating local topic information allows the model to comprehend the content of the article more deeply, resulting in high-quality summaries. When simultaneously integrating article hierarchical structure information and local topic information into the baseline model, the model leverages both as auxiliary information during summary generation. This leads to an improvement in summary quality. Comparing the results of Baseline (+ AHE) and Baseline (+ L-Inf) to Baseline (+ All), we observe an increase in all three metrics, indicating that each proposed module is necessary and contributes to the overall enhancement of the model’s performance.

**Table 7:** Ablation experiment on the PubMed dataset.

Dataset		PubMed	
Metrics models	R-1	R-2	R-L
Baseline	41.11	15.64	36.97
Baseline(**+ **AHE)	45.95	20.00	41.54
Baseline(+ L-Inf)	45.49	19.50	41.12
Baseline(+ All) (Our Model)	**46.49**	**20.52**	**42.06**

Significant values are in bold.

### Sentence position analysis

**Figure 3:**
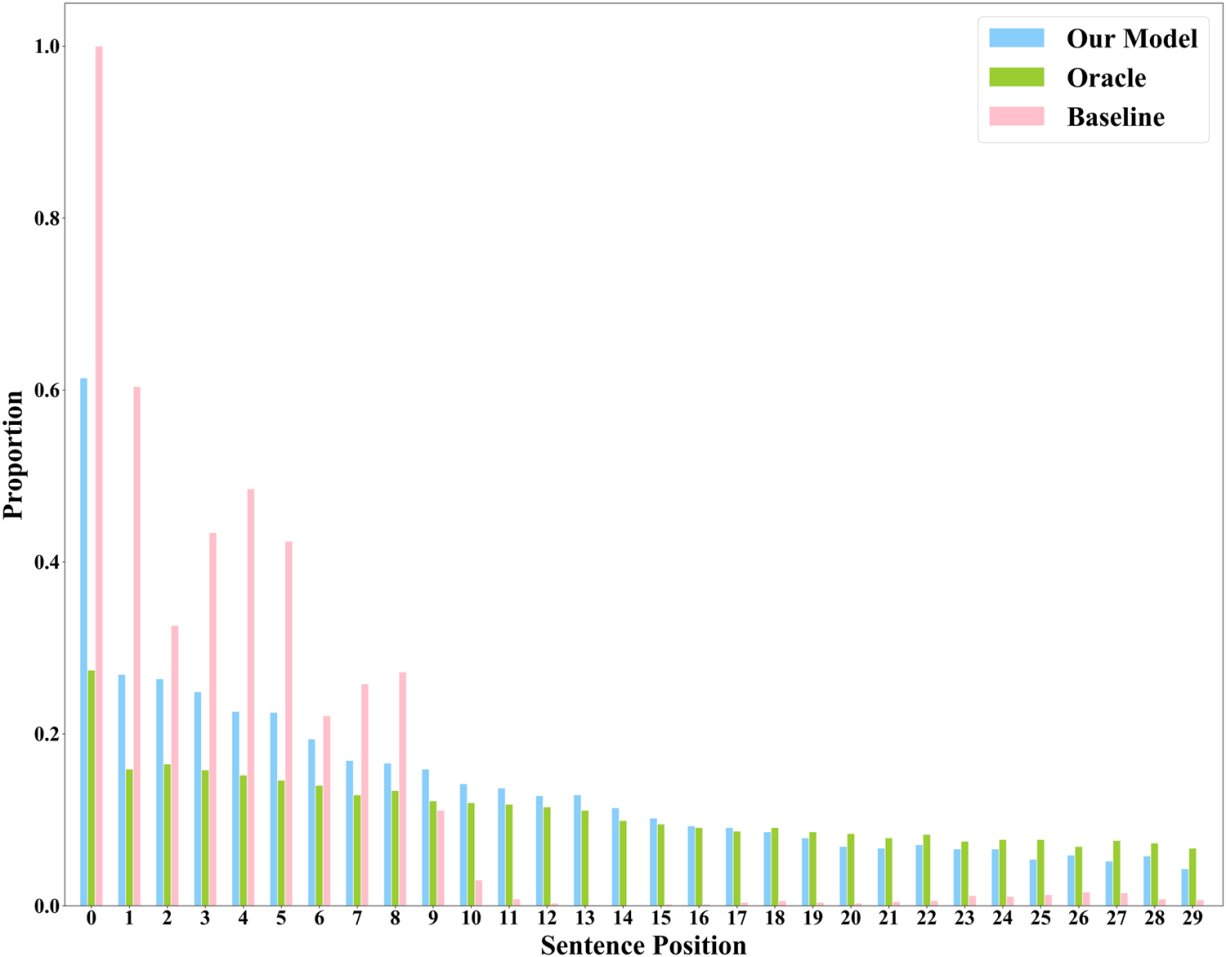
Distribution of summary sentences.

This study analyzed the location distribution of extract summary sentences in the source documents using different models on the PubMed testset. The results are shown in Fig. 3, where the X axis represents the sentence number and the Y axis represents the occurrence proportion (number of occurrences/total number of occurrences). We examine the distribution of the top 30 sentences extracted by our proposed model (blue), the Oracle method (green), and the baseline model (pink) across all documents in the PubMed testset. According to Fig. 3, it can be found that the summary distribution generated by the Oracle model is uniform. The baseline model lacks the perception ability of the overall document structure information and local topic information, leading to a bias towards extracting the first 10 sentences while ignoring the subsequent ones. In contrast, our proposed model overcomes the limitation that the baseline model only pays attention to the initial sentences. Additionally, the distribution of summary sentences generated by our model is close to that of Oracle. This indicates that by explicitly incorporating local topic information and article hierarchical structure information we proposed, the model gains a deeper understanding of the content in PubMed documents and successful learns the internal structure at a more meaningful level, effectively reducing its overreliance on the linear position of sentences.

## Conclusion

This paper mainly focuses on the impact of multiple topic information and the inherent hierarchical structure of long texts on the quality of model-generated summaries. To address the challenges posed by the abundance of topics and complex text hierarchy in generating summaries, this paper proposes a long text extractive summarization model that combines local topic information and text hierarchical structure information. Through comparative experiments conducted on the PubMed dataset, the results demonstrates superior performance in long text extractive summarization tasks compared to other models. Ablation experiments also confirm the necessity of each module proposed in this paper. Moreover, we recognize certain limitations in the model's extraction of hierarchical information from text. For instance, when processing news short texts like those in CNN/Daily Mail, which lack clear hierarchical structures, the model's enhancement effects are not significant. Therefore, in our future work, we plan to pay more attention to the topic information in the text. We aim to compare it with real summaries, construct comparative graph, and guide the model to choose sentences for summarization that are similar to those in real summaries.

## Data Availability

The PubMed dataset in this article is from open source links. Researchers in this field have integrated them, our PubMed dataset is available at https://github.com/QianRuan/histruct/releases/tag/data_and_models.
